# An improved composite ship magnetic field model with ellipsoid and magnetic dipole arrays

**DOI:** 10.1038/s41598-024-54848-6

**Published:** 2024-02-19

**Authors:** Binjie Lu, Xiaobing Zhang

**Affiliations:** 1 College of Weapons, Naval University of Engineering, Wuhan, 430033 Hubei China; 2 The 92279 Unit of the PLA, Qingdao, 266209 Shandong China

**Keywords:** Ship magnetic field, Composite model, Ellipsoid, Magnetic dipole array, Modeling accuracy, Coefficient matrix condition number, Computational complexity, Regularization methods, Electrical and electronic engineering, Computational science

## Abstract

In order to simultaneously maintain the ship magnetic field modeling accuracy, reduce the number of coefficient matrix conditions and the model computational complexity, an improved composite model is designed by introducing the magnetic dipole array model with a single-axis magnetic moment on the basis of the hybrid ellipsoid and magnetic dipole array model. First, the improved composite model of the ship's magnetic field is established based on the magnetic dipole array model with 3-axis magnetic moment, the magnetic dipole array model with only x-axis magnetic moment, and the ellipsoid model. Secondly, the set of equations for calculating the magnetic moments of the composite model is established, and for the problem of solving the pathological set of equations, the least-squares estimation, stepwise regression method, Tikhonov, and truncated singular value decomposition regularization methods are introduced in terms of the magnetic field, and generalized cross-validation is used to solve the optimal regularization parameters. Finally, a ship model test is designed to compare and analyze the effectiveness of the composite and hybrid models in four aspects: the number of coefficient matrix conditions of the model equation set, the relative error of magnetic field fitting, the relative error of magnetic field extrapolation, and the computational time complexity. The modeling results based on the ship model test data show that the composite model can be used for modeling the magnetic field of ships, and compared with the hybrid model, it reduces the number of coefficient matrix conditions and improves the computational efficiency on the basis of retaining a higher modeling accuracy, and it can be effectively applied in related scientific research and engineering.

## Introduction

Ship's magnetic field is the disturbed magnetic field generated by ferromagnetic ship disturbing the magnetic field around the earth, and there are four main sources of ship's magnetic field generation^1,2^. The finite element method for simulating and synthesizing ship magnetic field data has the advantage of high accuracy, but the disadvantage is that it needs to measure the complete envelope, which is difficult in practice and has high computational complexity^3,4^. The magnetic field equivalent source method is a simple and effective numerical calculation method for ship magnetic field reconstruction compared with the finite element method^5,6^ and boundary element method^7^, whose basic principle is to replace the original ferromagnetic objects by using several magnetic sources with analytical solutions, which is widely used because of its advantages of high stability, high accuracy and high computational efficiency^8^. And the equivalent source method can invert the model parameters based on the magnetic field measurements once the location of the magnetic sources is determined. Ship magnetic field equivalent source models include single magnetic dipole model^9–12^, magnetic monopole model^13^, magnetic dipole array model, multiple magnetic dipole model^5^, single ellipsoid model, hybrid ellipsoid and magnetic dipole array model^14^, and long ellipsoid harmonic model^15^. The single magnetic dipole model has fewer unknown parameters, less computational effort, and is suitable for far-field conditions, but cannot accurately simulate the target magnetic field in the near field. The hybrid ellipsoid and magnetic dipole array model simulates the target magnetic field with high accuracy and is suitable for near-field conditions.

In recent years, many scholars have carried out relevant research on ship magnetic field modeling. Jeung Giwoo presented an effective method for determining underwater magnetic field anomalies caused by residual magnetization of ferromagnetic hulls using a magnetic dipole modeling technique combined with material sensitivity analysis^16^. A. Sheinker used the finite element method and the LASSO algorithm to model the magnetic field based on 55 magnetic dipoles uniformly distributed on the surface of the ship model^5^. Kazimierz Jakubiuk used the Z-component of the ship's magnetic field to invert the parameters of the non-equally spaced magnetic dipole array model, including the magnetic dipole positions, magnetic dipole moments, and so on, and took into account the combined effect of fixed and induced magnetic moments^17^. Baowei He applied an iterative regularization algorithm based on conjugate gradient least squares based on the magnetic monopole model, showing that the algorithm can improve the inversion accuracy of ship magnetic field modeling^13^. Chengbao GUO adopts a three-dimensional magnetic monopole array model, constructs a system of linear equations for the inversion of a ship's magnetic source using the magnetic field difference, and solves the model parameters using the regularization method^18^. Mads Stormo Nilsson developed a long ellipsoidal harmonic model, which equates the magnetic field of a ship as a superposition of the magnetic fields of a set of magnetic multipoles placed in a long ellipsoidal coordinate system, and in order to improve the predictive power of the model and reduce overfitting, the model coefficients were solved using Lasso LARS^15^. JAROSLAW TARNAWSKI used virtual ellipsoid as a source of magnetic field data on the basis of multi-magnetic dipole model and superimposed noise to synthesize the magnetic field data, considered the intrinsic and induced magnetic moments of the magnetic target, laid the permanent magnetic dipole and the induced magnetic dipole in the same plane, respectively, and established the objective functions of magnetic dipole magnetic moments, positions, etc., and solved the optimization problem by using a nonlinear least-squares method^19^. Since then, JAROSLAW TARNAWSKI has utilized the model to conduct real ship sea trial tests, inverting the model parameters by Ridge and LASSO algorithms^20^. JAROSLAW TARNAWSKI investigated three-dimensional multi-dipole modeling by extending the training dataset to collect magnetic field data from all four cardinal directions and verified the robustness of the improved multi-dipole model using synthetic data after adding noise^21^. Jan-Ove Hall introduced an algorithm to decompose the intrinsic and induced magnetic fields, solved the magnetic field model using FEM, and gave the ship's magnetic field in the same region in different directions^22^. Miroslaw Woloszyn used a decomposition-based approach to calculate the magnetic field of each component of a magnetic target separately, where the overall magnetic characteristics of the object can be reproduced anywhere on the i.e., Earth, and validated the proposed method using finite element software^4^. In order to overcome the problem of the inability of the multi-magnetic dipole model in the literature^21^ to reproduce the magnetic field at unmeasured places, Miroslaw Woloszyn addresses the problem of reconstructing the magnetic field of a ship at an arbitrary position on the Earth by employing a multi-magnetic dipole model, constructing a nonlinear inversion model, inverting the intrinsic and induced magnetic moments using the nonlinear least-squares algorithm with trust-region reflective optimization(TRR), and predicting the magnetic field produced by a ship at an arbitrary position on the Earth using this model^23^.

The basic idea of modeling based on the hybrid model is to regard the magnetic dipole and ellipsoid as the magnetic field source of the ship, and by changing the magnitude, direction and number of magnetic moments of the magnetic dipole and ellipsoid, the magnetic field distribution consistent with the measured magnetic field can be generated. The magnetic field modeling problem can be regarded as an inverse problem, and the regularization method should be adopted when solving the inverse problem to avoid overfitting in the regression analysis, so as to prevent the magnetic dipoles with opposite directions from canceling each other^5^. Regularization methods include Tikhonov/Ridge, least absolute shrinkage and selection operator (LASSO), least angular regression (LARS), least square residual (LSMR), and conjugate gradient least squares (CGLS)^24–27^.

In order to simultaneously maintain the modeling accuracy and enhance the model computational efficiency, an improved composite model is designed on the basis of the hybrid model by introducing the magnetic dipole array model with a single-axis magnetic moment. First, the improved composite model of the ship's magnetic field is established based on the magnetic dipole array model with 3-axis magnetic moments, the magnetic dipole array model with only x-axis magnetic moments, and the ellipsoid model. Secondly, the set of equations for calculating the magnetic moments of the composite model is established, and the least squares estimation (LS), stepwise regression method, Tikhonov, and truncated singular value decomposition (TSVD) regularization methods are introduced with the magnetic field for the problem of solving the pathological set of equations, and the optimal regularization parameters are solved by using generalized cross-validation (GCV). Finally, ship model tests are designed to compare and analyze the effectiveness of the composite and hybrid models in four aspects, including the condition number of the coefficient matrix of the model equation set, the relative error of the magnetic field fitting, the relative error of the magnetic field extrapolation, and the computational time complexity. The modeling results based on the ship model test data show that the composite model can be used for modeling the magnetic field of the ship, and it improves the computational efficiency compared with the hybrid model while retaining a higher modeling accuracy.

## Improved composite equivalent source magnetic field model

Ship magnetic field modeling refers to the construction of some kind of model to accurately reflect the spatial distribution law of the ship magnetic field, and at the same time, it is necessary to extrapolate the measured magnetic field to the location of other depths, which can usually be equivalent to the ship as a hybrid model of ellipsoid and magnetic dipole array^14^, in order to further enhance the accuracy of magnetic field simulation on the ship while taking into account the complexity of the model, the introduction of a uniaxial magnetic moment on the basis of the hybrid model of the In order to further improve the magnetic field simulation accuracy of the ship while taking into account the model complexity, the magnetic dipole array model with uniaxial magnetic moment is introduced on the basis of the hybrid model, and the improved composite equivalent source magnetic field model of the ship is designed. Considering that the ship is in a stable and direct sailing state in a short period of time, the ship's intrinsic magnetic field and induced magnetic field can be studied together, so the intrinsic magnetic moment and induced magnetic moment of the magnetic dipole are no longer considered differently.

### General structure of the composite magnetic field model

**Figure 1:**
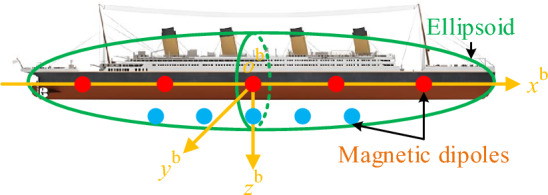
Improved composite magnetic field model for ships.

The improved composite equivalent source magnetic field model consists of 3 parts, part 1 is a magnetic dipole array model with a single-axis magnetic moment (x-direction), part 2 is a magnetic dipole array model with 3 axes, and part 3 is a single ellipsoid model as shown in Fig. 1. The ellipsoid is located in the center of the ship, and its long axis is equal to the length of the ship and the short axis is equal to the width of the ship, which is used to fit the macroscopic magnetic field of the ship. The magnetic dipole array with 3 axes is uniformly distributed on the ship's draft line to simulate the ship's localized inhomogeneous magnetic field. Magnetic dipole arrays with single-axis magnetic moments are located at a certain depth below the ship's draft line for fine-tuning the magnetic field. The carrier coordinate system (b-system) is established with the ship center as the origin, and the sensor coordinate system (s-system) is established with the sensor center as the origin. To simplify the representation, the variables under the b-system are not superscripted.

### Magnetic field calculation formulas

The corresponding magnetic field calculation formulas for the 3 models are as follows:

The magnetic field of a magnetic dipole array model with a 3-axis magnetic moment is calculated as

1 $$ \left[ {\begin{array}{*{20}c} {B_{x} } \\ {B_{y} } \\ {B_{z} } \\ \end{array} } \right] = \sum\limits_{i = 1}^{M} {\left[ {\begin{array}{*{20}c} {a_{x}^{i} } & {a_{y}^{i} } & {a_{z}^{i} } \\ {b_{x}^{i} } & {b_{y}^{i} } & {b_{z}^{i} } \\ {c_{x}^{i} } & {c_{y}^{i} } & {c_{z}^{i} } \\ \end{array} } \right]\left[ {\begin{array}{*{20}c} {m_{x}^{i} } \\ {m_{y}^{i} } \\ {m_{z}^{i} } \\ \end{array} } \right]} $$

where $${\mathbf{B}}_{{{\text{DA}}1}} = \left[ {\begin{array}{*{20}c} {B_{x} } & {B_{y} } & {B_{z} } \\ \end{array} } \right]^{{\text{T}}}$$ is the magnetic field vector of the magnetic dipole array model with 3-axis magnetic moments, and $m_{j}^{i} \left( {i = 1,2, \cdots ,M;j = x,y,z} \right)$ is the magnetic moment of the ith magnetic dipole j-axis in the b-series. The coefficients in the coefficient matrix are

2 $$ \left\{ \begin{gathered} a_{x}^{i} = \frac{{\mu_{0} }}{4\pi }\left( {\frac{3}{{\left\| {{\mathbf{r}}^{i} } \right\|_{2}^{5} }}x_{{}}^{i2} - \frac{1}{{\left\| {{\mathbf{r}}^{i} } \right\|_{2}^{3} }}} \right),a_{y}^{i} = \frac{{3\mu_{0} x^{i} y^{i} }}{{4\pi \left\| {{\mathbf{r}}^{i} } \right\|_{2}^{5} }},a_{z}^{i} = \frac{{3\mu_{0} x^{i} z^{i} }}{{4\pi \left\| {{\mathbf{r}}^{i} } \right\|_{2}^{5} }} \hfill \\ b_{x}^{i} = a_{y}^{i} ,b_{y}^{i} = \frac{{\mu_{0} }}{4\pi }\left( {\frac{3}{{\left\| {{\mathbf{r}}^{i} } \right\|_{2}^{5} }}y_{{}}^{i2} - \frac{1}{{\left\| {{\mathbf{r}}^{i} } \right\|_{2}^{3} }}} \right),b_{z}^{i} = \frac{{3\mu_{0} y^{i} z^{i} }}{{4\pi \left\| {{\mathbf{r}}^{i} } \right\|_{2}^{5} }} \hfill \\ c_{x}^{i} = a_{z}^{i} ,c_{y}^{i} = b_{z}^{i} ,c_{z}^{i} = \frac{{\mu_{0} }}{4\pi }\left( {\frac{3}{{\left\| {{\mathbf{r}}^{i} } \right\|_{2}^{5} }}z^{i2} - \frac{1}{{\left\| {{\mathbf{r}}^{i} } \right\|_{2}^{3} }}} \right) \hfill \\ \end{gathered} \right. $$

$\mu_{0} = 4\pi \times 10^{ - 7} {H \mathord{\left/ {\vphantom {H m}} \right. \kern-0pt} m}$ is the vacuum permeability, ${\varvec{r}}_{{}}^{i} = {\varvec{r}} - {\varvec{r}}_{{\text{d}}}^{i}$ is the coordinate of the magnetic sensor in the b-system relative to the ith magnetic dipole, $$\user2{r = }\left[ {\begin{array}{*{20}c} x & y & z \\ \end{array} } \right]^{{\text{T}}}$$ is the coordinate of the magnetic sensor in the b-system, $${\varvec{r}}_{{\text{d}}}^{i} = \left[ {\begin{array}{*{20}c} {\left( {i - {{\left( {M + 1} \right)} \mathord{\left/ {\vphantom {{\left( {M + 1} \right)} 2}} \right. \kern-0pt} 2}} \right)\Delta L} & 0 & 0 \\ \end{array} } \right]^{{\text{T}}}$$ is the coordinate of the ith magnetic dipole in the b-system, $\Delta L = {L \mathord{\left/ {\vphantom {L {\left( {M - 1} \right)}}} \right. \kern-0pt} {\left( {M - 1} \right)}}$ is the magnetic dipole spacing, and $L$ is the length of the ship.

The magnetic field of a magnetic dipole array model with a uniaxial magnetic moment is given by

3 $$ \left[ {\begin{array}{*{20}c} {B_{x} } \\ {B_{y} } \\ {B_{z} } \\ \end{array} } \right] = \sum\limits_{i = M + 1}^{M + N} {\left[ {\begin{array}{*{20}c} {a_{x}^{i} } \\ {b_{x}^{i} } \\ {c_{x}^{i} } \\ \end{array} } \right]m_{x}^{i} } $$

where $${\mathbf{B}}_{{{\text{DA2}}}} = \left[ {\begin{array}{*{20}c} {B_{x} } & {B_{y} } & {B_{z} } \\ \end{array} } \right]^{{\text{T}}}$$ is the magnetic field vector of the magnetic dipole array model with uniaxial magnetic moment, and $m_{x}^{i}$ is the magnetic moment of the ith magnetic dipole in the b-system. The coefficients in the coefficient matrix are $a_{x}^{i} = {{\mu_{0} \left( {3x_{{}}^{i2} - \left\| {{\mathbf{r}}^{i} } \right\|_{2}^{2} } \right)} \mathord{\left/ {\vphantom {{\mu_{0} \left( {3x_{{}}^{i2} - \left\| {{\mathbf{r}}^{i} } \right\|_{2}^{2} } \right)} {\left( {4\pi \left\| {{\mathbf{r}}^{i} } \right\|_{2}^{5} } \right)}}} \right. \kern-0pt} {\left( {4\pi \left\| {{\mathbf{r}}^{i} } \right\|_{2}^{5} } \right)}}$, $b_{x}^{i} = {{3\mu_{0} x^{i} y^{i} } \mathord{\left/ {\vphantom {{3\mu_{0} x^{i} y^{i} } {\left( {4\pi \left\| {{\mathbf{r}}^{i} } \right\|_{2}^{5} } \right)}}} \right. \kern-0pt} {\left( {4\pi \left\| {{\mathbf{r}}^{i} } \right\|_{2}^{5} } \right)}}$, $c_{x}^{i} = {{3\mu_{0} x^{i} z^{i} } \mathord{\left/ {\vphantom {{3\mu_{0} x^{i} z^{i} } {\left( {4\pi \left\| {{\mathbf{r}}^{i} } \right\|_{2}^{5} } \right)}}} \right. \kern-0pt} {\left( {4\pi \left\| {{\mathbf{r}}^{i} } \right\|_{2}^{5} } \right)}}$. ${\varvec{r}}^{i} = {\varvec{r}} - {\varvec{r}}_{{\text{d}}}^{i}$ is the coordinate of the magnetic sensor in the b-system with respect to the ith magnetic dipole, $${\varvec{r}}_{{\text{d}}}^{i} = \left[ {\begin{array}{*{20}c} {\left( {i - {{\left( {N + 1} \right)} \mathord{\left/ {\vphantom {{\left( {N + 1} \right)} 2}} \right. \kern-0pt} 2}} \right)\Delta L^{\prime} } & 0 & {z_{{\text{d}}} } \\ \end{array} } \right]^{{\text{T}}}$$ is the coordinate of the ith magnetic dipole in the b-system, $\Delta L^{\prime}$ is the magnetic dipole spacing, and $z_{{\text{d}}}$ is the distance between the magnetic dipole and the draft.

The ellipsoidal magnetic field is given by

4 $$ \left[ {\begin{array}{*{20}c} {B_{x} } \\ {B_{y} } \\ {B_{z} } \\ \end{array} } \right] = \left[ {\begin{array}{*{20}c} {a_{x}^{M + N + 1} } & {a_{y}^{M + N + 1} } & {a_{z}^{M + N + 1} } \\ {b_{x}^{M + N + 1} } & {b_{y}^{M + N + 1} } & {b_{z}^{M + N + 1} } \\ {c_{x}^{M + N + 1} } & {c_{y}^{M + N + 1} } & {c_{z}^{M + N + 1} } \\ \end{array} } \right]\left[ {\begin{array}{*{20}c} {m_{x}^{M + N + 1} } \\ {m_{y}^{M + N + 1} } \\ {m_{z}^{M + N + 1} } \\ \end{array} } \right] $$

where $${\mathbf{B}}_{{\text{E}}} = \left[ {\begin{array}{*{20}c} {B_{x} } & {B_{y} } & {B_{z} } \\ \end{array} } \right]^{{\text{T}}}$$ is the magnetic field vector of the ellipsoid and $m^{M + N + 1}$ is the magnetic moment of the ellipsoid. The coefficients in the coefficient matrix are

5 $$ \left\{ \begin{gathered} a_{x}^{i} = \frac{{3\mu_{0} }}{4\pi }\left( {\frac{{A_{{}}^{i} }}{{K^{2} t_{{}}^{i} }} + \frac{1}{{2K^{3} }}\ln \frac{{A_{{}}^{i} - K}}{{A_{{}}^{i} + K}}} \right),a_{y}^{i} = \frac{{3\mu_{0} }}{4\pi }\frac{{x_{{}}^{i} y_{{}}^{i} }}{{A_{{}}^{i} B_{{}}^{i2} t_{{}}^{i} }},a_{z}^{i} = \frac{{3\mu_{0} }}{4\pi }\frac{{x_{{}}^{i} z_{{}}^{i} }}{{A_{{}}^{i} B_{{}}^{i2} t_{{}}^{i} }} \hfill \\ b_{x}^{i} = a_{y}^{i} ,b_{y}^{i} = \frac{{3\mu_{0} }}{8\pi }\left( {\frac{{2A_{{}}^{i} y_{{}}^{i2} }}{{B_{{}}^{i4} t_{{}}^{i} }} - \frac{{A_{{}}^{i} }}{{B_{{}}^{i2} K^{2} }} - \frac{1}{{2K^{3} }}\ln \frac{{A_{{}}^{i} - K}}{{A_{{}}^{i} + K}}} \right),b_{z}^{i} = \frac{{3\mu_{0} }}{4\pi }\frac{{A_{{}}^{i} y_{{}}^{i} z_{{}}^{i} }}{{B_{{}}^{i4} t_{{}}^{i} }} \hfill \\ c_{x}^{i} = a_{z}^{i} ,c_{y}^{i} = b_{z}^{i} ,c_{z}^{i} = \frac{{3\mu_{0} }}{8\pi }\left( {\frac{{2A_{{}}^{i} z_{{}}^{i2} }}{{B_{{}}^{i4} t_{{}}^{i} }} - \frac{{A_{{}}^{i} }}{{B_{{}}^{i2} K^{2} }} - \frac{1}{{2K^{3} }}\ln \frac{{A_{{}}^{i} - K}}{{A_{{}}^{i} + K}}} \right) \hfill \\ t_{{}}^{i} = \sqrt {\left( {\left\| {{\mathbf{r}}^{i} } \right\|_{2}^{2} + K^{2} } \right)^{2} - 4K^{2} x_{{}}^{i2} } ,K = \sqrt {\left( {{L \mathord{\left/ {\vphantom {L 2}} \right. \kern-0pt} 2}} \right)^{2} - \left( {{W \mathord{\left/ {\vphantom {W 2}} \right. \kern-0pt} 2}} \right)^{2} } \hfill \\ A_{{}}^{i} = \sqrt {\frac{1}{2}\left( {\left\| {{\mathbf{r}}^{i} } \right\|_{2}^{2} + K^{2} + t_{{}}^{i} } \right)} \hfill \\ B_{{}}^{i} = \sqrt {\frac{1}{2}\left( {\left\| {{\mathbf{r}}^{i} } \right\|_{2}^{2} - K^{2} + t_{{}}^{i} } \right)} \hfill \\ \end{gathered} \right. $$

$L$ is the length of the ship and $W$ is the width of the ship.

The composite magnetic field model magnetic field ${\varvec{B}}_{{\text{C}}}$ is calculated as

6 $$ {\varvec{B}}_{{\text{C}}} = {\mathbf{B}}_{{{\text{DA}}1}} + {\mathbf{B}}_{{\text{E}}} + {\mathbf{B}}_{{{\text{DA2}}}} = {\mathbf{F}}_{{\text{C}}} {\mathbf{m}}_{{\text{C}}} $$

where the magnetic moments ${\mathbf{m}}_{{\text{C}}}$ of the magnetic dipole and ellipsoid are

7 $$ {\mathbf{m}}_{{\text{C}}} \user2{ = }\left[ {\begin{array}{*{20}c} {m_{x}^{1} } & {m_{y}^{1} } & {m_{z}^{1} } & \cdots & {m_{x}^{M} } & {m_{y}^{M} } & {m_{z}^{M} } & {m_{x}^{M + 1} } & \cdots & {m_{x}^{M + N} } & {m_{x}^{M + N + 1} } & {m_{y}^{M + N + 1} } & {m_{z}^{M + N + 1} } \\ \end{array} } \right]^{\rm T} $$

The coefficient matrix ${\mathbf{F}}_{{\text{C}}}$ is

8 $$ {\mathbf{F}}_{{\text{C}}} = \left[ {\begin{array}{*{20}c} {a_{x}^{1} } & {a_{y}^{1} } & {a_{z}^{1} } & \cdots & {a_{x}^{M} } & {a_{y}^{M} } & {a_{z}^{M} } & {a_{x}^{M + 1} } & \cdots & {a_{x}^{M + N} } & {a_{x}^{M + N + 1} } & {a_{y}^{M + N + 1} } & {a_{z}^{M + N + 1} } \\ {b_{x}^{1} } & {b_{y}^{1} } & {b_{z}^{1} } & \cdots & {b_{x}^{M} } & {b_{y}^{M} } & {b_{z}^{M} } & {b_{x}^{M + 1} } & \cdots & {b_{x}^{M + N} } & {b_{x}^{M + N + 1} } & {b_{y}^{M + N + 1} } & {b_{z}^{M + N + 1} } \\ {c_{x}^{1} } & {c_{y}^{1} } & {c_{z}^{1} } & \cdots & {c_{x}^{M} } & {c_{y}^{M} } & {c_{z}^{M} } & {c_{x}^{M + 1} } & \cdots & {c_{x}^{M + N} } & {c_{x}^{M + N + 1} } & {c_{y}^{M + N + 1} } & {c_{z}^{M + N + 1} } \\ \end{array} } \right] $$

where $i = 1, \cdots ,M$ corresponds to the magnetic dipole array model with a 3-axis magnetic moment, to the magnetic dipole array model with a single-axis magnetic moment, and $i = M + 1, \cdots ,M + N$ corresponds to the ellipsoid model.

## Composite magnetic field model magnetic moment calculation method

The problem of calculating the magnetic moment of a ship's magnetic field model can be regarded as a problem of solving a system of overdetermined Equations. ^14^. For the problem of solving the overdetermined equations, the least squares estimation, stepwise regression method^14^, Tikhonov-GCV, and TSVD-GCV regularization methods are used to solve the problem, respectively.

### Equations for solving magnetic moment

**Figure 2:**
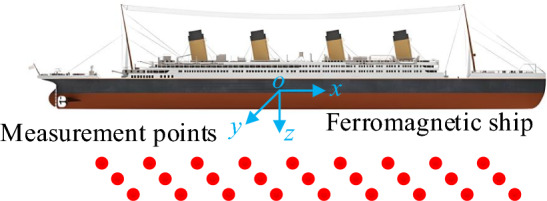
Schematic diagram of ship's magnetic field measurement.

A schematic diagram of the magnetic field measurement at a certain depth plane below the ship is shown in Fig. 2. In the actual magnetic field measurement, the measurement data will be interfered by noise, so the system of equations for solving the magnetic moment under the b-system is

9 $$ {\mathbf{B}} = {\mathbf{Fm}} + {\mathbf{e}} $$

where $${\mathbf{B}} = \left[ {\begin{array}{*{20}c} {B_{x1}^{{}} } & \cdots & {B_{xN}^{{}} } & {B_{y1}^{{}} } & \cdots & {B_{yN}^{{}} } & {B_{z1}^{{}} } & \cdots & {B_{zN}^{{}} } \\ \end{array} } \right]^{{\text{T}}}$$ is the magnetic field at N measurement points in the carrier coordinate system and the observation noise is Gaussian white noise, ${\mathbf{e}} \sim N\left( {{\mathbf{0}},{\mathbf{R}}} \right)$. The coefficient matrix ${\mathbf{F}}$ is

10 $$ {\mathbf{F}} = \left[ {\begin{array}{*{20}c} {a_{x1}^{1} } & {a_{y1}^{1} } & {a_{z1}^{1} } & \cdots & {a_{x1}^{M} } & {a_{y1}^{M} } & {a_{z1}^{M} } & {a_{x1}^{M + 1} } & \cdots & {a_{x1}^{M + N} } & {a_{x1}^{M + N + 1} } & {a_{y1}^{M + N + 1} } & {a_{z1}^{M + N + 1} } \\ \vdots & \vdots & \vdots & \cdots & \vdots & \vdots & \vdots & \vdots & \cdots & \vdots & \vdots & \vdots & \vdots \\ {a_{xN}^{{1}} } & {a_{yN}^{{1}} } & {a_{zN}^{{1}} } & \cdots & {a_{xN}^{M} } & {a_{yN}^{M} } & {a_{zN}^{M} } & {a_{xN}^{M + 1} } & \cdots & {a_{xN}^{M + N} } & {a_{xN}^{M + N + 1} } & {a_{yN}^{M + N + 1} } & {a_{zN}^{M + N + 1} } \\ {b_{x1}^{1} } & {b_{y1}^{1} } & {b_{z1}^{1} } & \cdots & {b_{x1}^{M} } & {b_{y1}^{M} } & {b_{z1}^{M} } & {b_{x1}^{M + 2} } & \cdots & {b_{x1}^{M + N} } & {b_{x1}^{M + N + 1} } & {b_{y1}^{M + N + 1} } & {b_{z1}^{M + N + 1} } \\ \vdots & \vdots & \vdots & \cdots & \vdots & \vdots & \vdots & \vdots & \vdots & \vdots & \vdots & \vdots & \vdots \\ {b_{xN}^{{1}} } & {b_{yN}^{{1}} } & {b_{zN}^{{1}} } & \cdots & {b_{xN}^{M} } & {b_{yN}^{M} } & {b_{zN}^{M} } & {b_{xN}^{M + 1} } & \cdots & {b_{xN}^{M + N} } & {b_{xN}^{M + N + 1} } & {b_{yN}^{M + N + 1} } & {b_{zN}^{M + N + 1} } \\ {c_{x1}^{1} } & {c_{y1}^{1} } & {c_{z1}^{1} } & \cdots & {c_{x1}^{M} } & {c_{y1}^{M} } & {c_{z1}^{M} } & {c_{x1}^{M + 1} } & \cdots & {c_{x1}^{M + N} } & {c_{x1}^{M + N + 1} } & {c_{y1}^{M + N + 1} } & {c_{z1}^{M + N + 1} } \\ \vdots & \vdots & \vdots & \cdots & \vdots & \vdots & \vdots & \vdots & \cdots & \vdots & \vdots & \vdots & \vdots \\ {c_{xN}^{{1}} } & {c_{yN}^{{1}} } & {c_{zN}^{{1}} } & \cdots & {c_{xN}^{M} } & {c_{yN}^{M} } & {c_{zN}^{M} } & {c_{xN}^{M + 1} } & \cdots & {c_{xN}^{M + N} } & {c_{xN}^{M + N + 1} } & {c_{yN}^{M + N + 1} } & {c_{zN}^{M + N + 1} } \\ \end{array} } \right] \in {\mathbb{R}}^{{3N \times \left( {3M + N + 3} \right)}} $$

where the coefficients are calculated according to Eqs. (2), (3) and (5) and the magnetic moments are the same as Eq. (7).

### Least squares estimation

Considering the equations for solving the magnetic moment can be reduced to the following observation equation

11 $$ {\mathbf{y}} = {\mathbf{Ax}} + {\mathbf{e}} $$

where ${\mathbf{y}} \in {\mathbb{R}}^{m}$, ${\mathbf{A}} \in {\mathbb{R}}^{m \times n}$, ${\mathbf{x}} \in {\mathbb{R}}^{m}$, ${\mathbf{e}} \in {\mathbb{R}}^{m}$, assuming that the observation noise is Gaussian white noise, ${\mathbf{e}} \sim N\left( {{\mathbf{0}},{\mathbf{R}}} \right)$. The singular value decomposition of ${\mathbf{A}}$ is given by

12 $$ {\mathbf{A}} = {\mathbf{U\Sigma V}}^{{\text{T}}} $$

where ${\mathbf{U}} \in {\mathbb{R}}^{m \times n}$, ${\mathbf{V}} \in {\mathbb{R}}^{n \times n}$ is the unit orthogonal matrix, $${{\varvec{\Sigma}}} = {\text{diag}}\left( {\left[ {\begin{array}{*{20}c} {\lambda_{1} } & \cdots & {\lambda_{n} } \\ \end{array} } \right]} \right) \in {\mathbb{R}}^{n \times n}$$ is the diagonal matrix, and $\lambda_{1} \ge \lambda_{2} \cdots \ge \lambda_{n}$.

The linear model in Eq. (11) satisfies the Gauss–Markov theorem, and the optimal estimate of the magnetic moment is the optimal linear unbiased estimation (BLUE)^5^

13 $$ {\hat{\mathbf{x}}} = \left( {{\mathbf{A}}^{{\text{T}}} {\mathbf{R}}^{ - 1} {\mathbf{A}}} \right)^{ - 1} {\mathbf{A}}^{{\text{T}}} {\mathbf{R}}^{ - 1} {\mathbf{y}} $$

The spectral decomposition of LS estimation for unknown parameters takes the form^25^

14 $$ {\hat{\mathbf{x}}}_{{{\text{LS}}}} = \sum\limits_{i = 1}^{n} {\frac{{{\mathbf{u}}_{i}^{{\text{T}}} {\mathbf{y}}}}{{\lambda_{i} }}{\mathbf{v}}_{i} } = \sum\limits_{i = 1}^{n} {\left( {{\mathbf{v}}_{i}^{{\text{T}}} {\mathbf{x}} + \frac{{{\mathbf{u}}_{i}^{{\text{T}}} {\mathbf{e}}}}{{\lambda_{i} }}} \right){\mathbf{v}}_{i} } $$

where ${\mathbf{u}}_{i} \in {\mathbb{R}}^{m}$ and ${\mathbf{v}}_{i} \in {\mathbb{R}}^{n}$ are the ith column vectors of ***U*** and ***V***, respectively. The mean square error of the least squares estimation is

15 $$ {\text{MSE}}\left( {{\hat{\mathbf{x}}}_{{{\text{LS}}}} } \right) = {\mathbf{R}}\sum\limits_{i = 1}^{n} {\frac{{{\mathbf{v}}_{i} {\mathbf{v}}_{i}^{{\text{T}}} }}{{\lambda_{i}^{{2}} }}} $$

The trace of ${\text{MSE}}\left( {{\hat{\mathbf{x}}}_{{{\text{LS}}}} } \right)$ is

16 $$ {\text{Tr}}\left[ {{\text{MSE}}\left( {{\hat{\mathbf{x}}}_{{{\text{LS}}}} } \right)} \right] = \sum\limits_{i = 1}^{n} {\frac{{\mathbf{R}}}{{\lambda_{i}^{{2}} }}} $$

The trace of ${\text{MSE}}\left( {{\hat{\mathbf{x}}}_{{{\text{LS}}}} } \right)$ reflects the difference between the estimate and the true value. When the condition number *λ*_max_/*λ*_min_ is very large, the LS estimates are susceptible to noise leading to unstable solutions.

### Tikhonov regularization

The Tikhonov regularization method filters or attenuates small singular values to retain all the information, and the Tikhonov regularization takes the form^26^

17 $$ \mathop {\min }\limits_{{\mathbf{x}}} \left\| {{\mathbf{Ax}} - {\mathbf{y}}} \right\|_{2}^{2} + \alpha^{2} \left\| {{\mathbf{Lx}}} \right\|_{2}^{2} $$

where $\alpha$ is the regularization parameter and ${\mathbf{L}}$ is the regularization matrix. The solution of the regularization estimation is

18 $$ {\mathbf{x}}_{\alpha } = \left( {{\mathbf{A}}^{{\text{T}}} {\mathbf{A}} + \alpha^{2} {\mathbf{I}}} \right)^{ - 1} {\mathbf{A}}^{{\text{T}}} {\mathbf{y}} $$

The spectral decomposition of the Tikhonov regularized estimate is of the form^25^

19 $$ {\hat{\mathbf{x}}}_{{{\text{Tikh}}}} = \sum\limits_{i = 1}^{n} {\frac{{\lambda_{i}^{{2}} }}{{\lambda_{i}^{{2}} + \alpha }}\frac{{{\mathbf{u}}_{i}^{{\text{T}}} {\mathbf{y}}}}{{\lambda_{i} }}{\mathbf{v}}_{i} } = \sum\limits_{i = 1}^{n} {\left( {\frac{{\lambda_{i}^{{2}} }}{{\lambda_{i}^{{2}} + \alpha }}{\mathbf{v}}_{i}^{{\text{T}}} {\mathbf{x}} + \frac{{\lambda_{i}^{{2}} }}{{\lambda_{i}^{{2}} + \alpha }}\frac{{{\mathbf{u}}_{i}^{{\text{T}}} {\mathbf{e}}}}{{\lambda_{i} }}} \right){\mathbf{v}}_{i} } $$

where α is the regularization parameter. The mean square error of the Tikhonov regularization estimate is

20 $$ {\text{MSE}}\left( {{\hat{\mathbf{x}}}_{{{\text{Tikh}}}} } \right) = {\mathbf{R}}\sum\limits_{i = 1}^{n} {\frac{{\lambda_{i}^{{2}} {\mathbf{v}}_{i} {\mathbf{v}}_{i}^{{\text{T}}} }}{{\left( {\lambda_{i}^{{2}} + \alpha } \right)^{2} }}} + \left( {\sum\limits_{i = 1}^{n} {\frac{{\alpha {\mathbf{v}}_{i}^{{\text{T}}} {\mathbf{x}}}}{{\lambda_{i}^{{2}} + \alpha }}{\mathbf{v}}_{i} } } \right)\left( {\sum\limits_{i = 1}^{n} {\frac{{\alpha {\mathbf{v}}_{i}^{{\text{T}}} {\mathbf{x}}}}{{\lambda_{i}^{{2}} + \alpha }}{\mathbf{v}}_{i}^{{\text{T}}} } } \right) $$

The trace of ${\text{MSE}}\left( {{\hat{\mathbf{x}}}_{{{\text{Tikh}}}} } \right)$ is

21 $$ {\text{Tr}}\left[ {{\text{MSE}}\left( {{\hat{\mathbf{x}}}_{{{\text{Tikh}}}} } \right)} \right] = \sum\limits_{i = 1}^{n} {\left[ {\frac{{\lambda_{i}^{{2}} {\mathbf{R}}}}{{\left( {\lambda_{i}^{{2}} + \alpha } \right)^{2} }} + \frac{{\alpha^{2} \left( {{\mathbf{v}}_{i}^{{\text{T}}} {\mathbf{x}}} \right)^{2} }}{{\left( {\lambda_{i}^{{2}} + \alpha } \right)^{2} }}} \right]} $$

Too small a value of the regularization parameter α tends to lead to under-regularization and vice versa for over-regularization.

### Truncated singular value decomposition

Removing the n-k components of the least-squares estimate in the hyperspectral domain, the spectral decomposition of the TSVD estimate takes the form^25^

22 $$ {\hat{\mathbf{x}}}_{{{\text{TSVD}}}} = \sum\limits_{i = 1}^{k} {\frac{{{\mathbf{u}}_{i}^{{\text{T}}} {\mathbf{y}}}}{{\lambda_{i} }}{\mathbf{v}}_{i} } = \sum\limits_{i = 1}^{k} {\left( {{\mathbf{v}}_{i}^{{\text{T}}} {\mathbf{x}} + \frac{{{\mathbf{u}}_{i}^{{\text{T}}} {\mathbf{e}}}}{{\lambda_{i} }}} \right){\mathbf{v}}_{i} } $$

The mean square error of the TSVD estimation is

23 $$ {\text{MSE}}\left( {{\hat{\mathbf{x}}}_{{{\text{TSVD}}}} } \right) = {\mathbf{R}}\sum\limits_{i = 1}^{n} {\frac{{{\mathbf{v}}_{i} {\mathbf{v}}_{i}^{{\text{T}}} }}{{\lambda_{i}^{{2}} }}} + \left( {\sum\limits_{i = k + 1}^{n} {{\mathbf{v}}_{i}^{{\text{T}}} {\mathbf{xv}}_{i} } } \right)\left( {\sum\limits_{i = k + 1}^{n} {{\mathbf{v}}_{i}^{{\text{T}}} {\mathbf{xv}}_{i}^{{\text{T}}} } } \right) $$

The trace of ${\text{MSE}}\left( {{\hat{\mathbf{x}}}_{{{\text{TSVD}}}} } \right)$ is

24 $$ {\text{Tr}}\left[ {{\text{MSE}}\left( {{\hat{\mathbf{x}}}_{{{\text{TSVD}}}} } \right)} \right] = \sum\limits_{i = 1}^{k} {\frac{{\mathbf{R}}}{{\lambda_{i}^{{2}} }}} + \sum\limits_{i = k + 1}^{n} {\left( {{\mathbf{v}}_{i}^{{\text{T}}} {\mathbf{x}}} \right)^{2} } $$

Too small a value of the truncation parameter k tends to lead to over-regularization and vice versa for under-regularization.

### Regularized parameter optimization methods

Regularization parameter optimization methods include Discrepancy Principle^28^, L-curve^29^, GCV^30^, NCP^28^, etc. L-curve is a logarithmic graph that characterizes the relationship between and change in terms of α parameter, and the approximation of the optimal regularization parameter can be computed by locating the corners of the L-curve, and for the continuous L-curve, the corner is the point with maximum curvature on the L-curve of the logarithmic scaled L-curve at the point with maximum curvature on the L-curve^31^. However, the L-curve method is not applicable in the case of no noise or very low noise^28^. The goal of GCV is to find the value of α such that the data can be predicted as accurately as possible. The Discrepancy Principle relies on the accurate estimation of the error paradigm. The GCV aims to reduce the prediction error, and it is a more robust method. The NCP is a statistically based method, which may misidentify low-frequency noise as signals. low-frequency noise as a signal, which leads to unsmoothing.

For the magnetic field model magnetic moment solution problem, GCV is used for parameter optimization. The GCV equations are^26,32^

25 $$ G_{{{\mathbf{A}},{\mathbf{y}}}} \left( \alpha \right) = \frac{{\left\| {\left( {{\mathbf{I}} - {\mathbf{AA}}_{\alpha }^{\dag } } \right){\mathbf{x}}_{\alpha } } \right\|_{2}^{2} }}{{\left[ {{\text{Tr}}\left( {{\mathbf{I}} - {\mathbf{AA}}_{\alpha }^{\dag } } \right)} \right]^{2} }} $$

where ${\mathbf{A}}_{\alpha }^{\dag } = \left( {{\mathbf{A}}^{{\text{T}}} {\mathbf{A}} + \alpha^{2} {\mathbf{I}}} \right)^{ - 1} {\mathbf{A}}^{{\text{T}}}$. From the Scherman-Morrison-Woodburg theorem we have

26 $$ \alpha \left( {{\mathbf{A}}^{{\text{T}}} {\mathbf{A}} + \alpha {\mathbf{I}}} \right)^{ - 1} = {\mathbf{I}} - {\mathbf{A}}\left( {{\mathbf{A}}^{{\text{T}}} {\mathbf{A}} + \alpha {\mathbf{I}}} \right)^{ - 1} {\mathbf{A}}^{{\text{T}}} $$

Substituting Eq. (26) into Eq. (25), we get

27 $$ G_{{{\mathbf{A}},{\mathbf{y}}}} \left( \alpha \right) = \frac{{\left\| {\left( {{\mathbf{A}}^{{\text{T}}} {\mathbf{A}} + \alpha {\mathbf{I}}} \right)^{ - 1} {\mathbf{x}}_{\alpha } } \right\|_{2}^{2} }}{{\left[ {{\text{Tr}}\left( {{\mathbf{A}}^{{\text{T}}} {\mathbf{A}} + \alpha {\mathbf{I}}} \right)} \right]^{2} }} $$

Thus, the optimal regularization parameter is

28 $$ \alpha = \arg \min \left\{ {G_{{{\mathbf{A}},{\mathbf{y}}}} \left( \alpha \right)} \right\} $$

**Figure 3:**
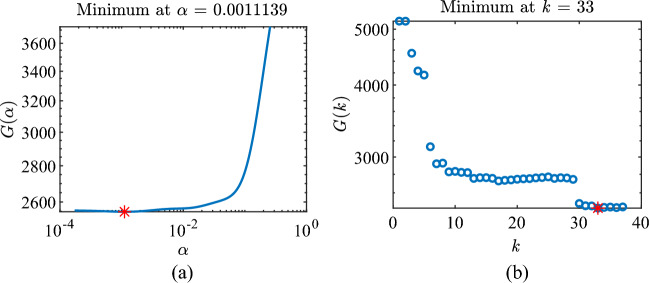
Determination of regularization parameters using the GCV approach. (**a**) α of Tikhonov, (**b**) k of TSVD.

The selection of regularization parameters α and k using the GCV method is shown in Fig. 3.

## Ship model test validation

### Ship model test setup

**Figure 4:**
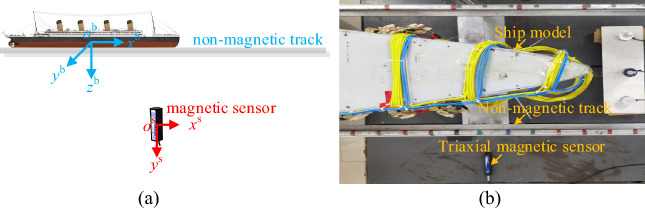
Ship model test system. (**a**) Schematic diagram of the test system, (**b**) Measuring devices.

The test uses a scaling-down ship model, whose dimensional parameters are the dimensions of the real ship corresponding to the ship model, not the dimensions of the ship model itself, and the depths, positive transits, and trajectories are the measured dimensions of the real ship corresponding to the ship after conversion. The ship model test measured the magnetic fields of 3 tracks at each of the 2 depths, as shown in Table 1. Three measurement lines were uniformly set below the model at a distance of 1W and 2W, with z-coordinates of 1W and 2W, and y-coordinates of − 0.5 W, 0, and 0.5 W, which corresponded to the port, keel, and starboard sides of the ship model, respectively. There are 81 measurement points in each measurement line, with x coordinates of − 1.5 L–1.5 L (L denotes the length of the ship, W denotes the width of the ship). The magnetic sensor is Mag-13MSL100, and the position is shown in Fig. 4. The single-point magnetic field measurement is used to move the ship on the track with equal spacing, and every time the ship moves, a period of time is measured, and the average value of the period is calculated as the corresponding magnetic field value of the measurement point, and the magnetic field value of each point is connected to form a continuous ship magnetic field through the characteristics. The following is the magnetic field modeling of the ship model based on the composite model and the magnetic field measurement data.

**Table 1:** Ship model test conditions.

Length (m)	Width (m)	*z* (m)	*y* (m)	*x* (m)
153	17.3	17.2	− 8.64	− 128:3.2:128
			0	
			8.64	
		28.8	− 8.64	
			0	
			8.64

### Improved composite model versus traditional hybrid model setup

**Figure 5:**
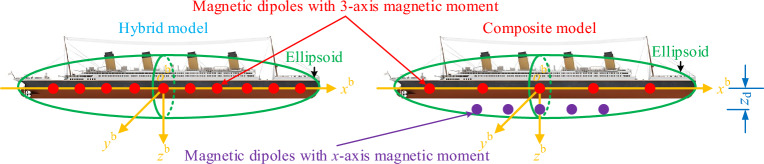
Comparison of the two models.

The parameters of the improved composite model and the conventional hybrid model^33^ are shown in Table 2. In order to compare the two models in terms of modeling accuracy of the ship's magnetic field, the number of coefficient matrix conditions and other factors, the same number of magnetic dipoles and the number of ellipsoids are used. For the large ship model used in the test, the number of magnetic dipoles needs to take a higher number to ensure the modeling accuracy^34^, and the ellipsoids of the two models have the same size and the same coordinates in the b-system. The difference between the two models is shown in Fig. 5. In the hybrid model, the spacing of the magnetic dipoles is calculated based on the ship length. In the composite model, the spacing of the 3-axis magnetic moment magnetic dipole is calculated according to the length of the ship, and the spacing and coordinates *z*_d_ of the x-axis magnetic moment magnetic dipole are adjusted according to the dimensions of the ship.

**Table 2:** Improved composite and conventional hybrid model parameters.

Model	Ellipsoid number	Number of magnetic dipoles with 3-axis magnetic moments	Number of magnetic dipoles with x-axis magnetic moments
Hybrid	1	15	0
Composite	1	10	5

### Model evaluation indicators

(1) The coefficient matrix condition number is

29 $$ Cond\left( {\mathbf{F}} \right) = \left\| {\mathbf{F}} \right\|_{2} \cdot \left\| {{\mathbf{F}}^{ - 1} } \right\|_{2} $$

The condition number is related to b only and reflects the effect of the original data on the solution. If the condition number is larger, the system of equations is more pathological and the solution is less robust.

(2) The relative error of the magnetic field fit is

30 $$ E_{1} = \frac{{\left\| {{\mathbf{F\hat{m}}} - {\mathbf{B}}} \right\|_{2} }}{{\left\| {\mathbf{B}} \right\|_{2} }} $$

where ${\mathbf{F}}$ is the coefficient matrix, ${\mathbf{F\hat{m}}}$ is the simulated magnetic field for solving the magnetic moment calculation, and ${\mathbf{B}}$ is the measured magnetic field without noise.

(3) The relative error of the magnetic field extrapolation is

31 $$ E_{2} = \frac{{\left\| {{\mathbf{F}}^{\prime} {\hat{\mathbf{m}}} - {\mathbf{B}}^{\prime} } \right\|_{2} }}{{\left\| {{\mathbf{B}}^{\prime} } \right\|_{2} }} $$

where ${\mathbf{F}}^{\prime}$ is the matrix of coefficients in the other plane, ${\mathbf{F}}^{\prime} {\hat{\mathbf{m}}}$ is the in-plane magnetic field in the other plane for solving the magnetic moment calculation, and ${\mathbf{B}}^{\prime}$ is the noise-free measured magnetic field in the other plane.

(4) CPU computation time is the time required for the algorithm to complete the magnetic moment estimation. The computer processor is 11th Gen Intel(R) Core (TM) i5-1155G7 @ 2.50 GHz with 16.0 GB of RAM.

### Comparative analysis of models

**Figure 6:**
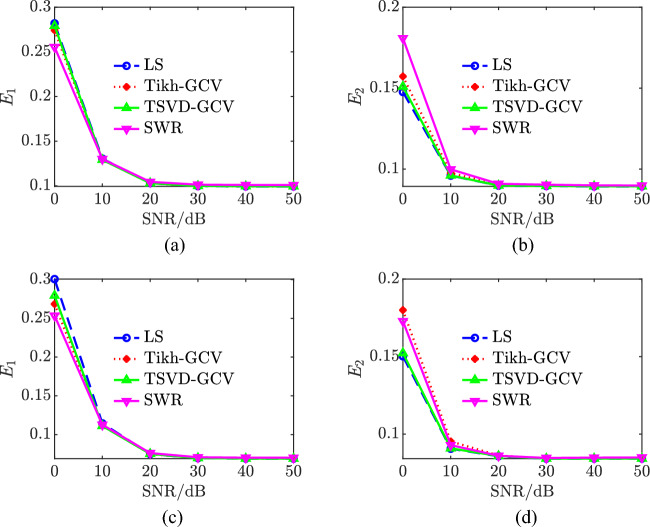
Comparison of relative errors of magnetic field fitting and relative errors of magnetic field extrapolation for two models. (**a**) Relative error of magnetic field fitting for composite model, (**b**) Composite model magnetic field extrapolation relative error, (**c**) Relative error of the hybrid model magnetic field fit and (**d**) Relative errors in extrapolation of hybrid model magnetic fields.

**Figure 7:**
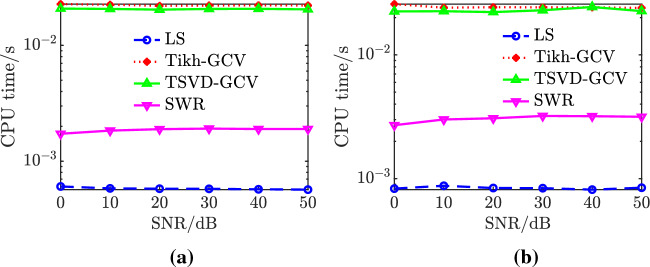
CPU computation time. (**a**) Composite model, (**b**) Hybrid model.

**Figure 8:**
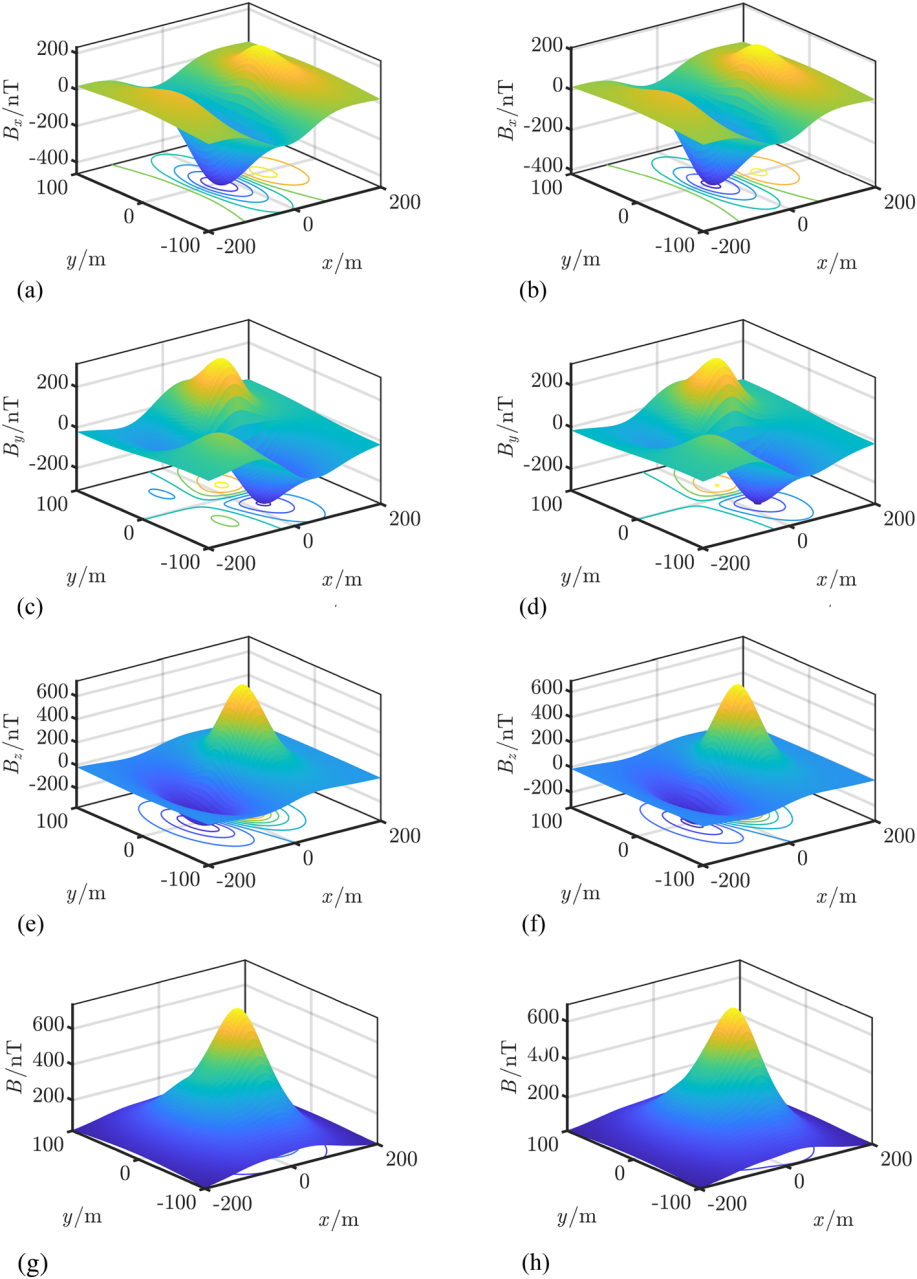
Spatial distribution of magnetic field based on TSVD-GCV algorithm solution(SNR = 0 dB, *z* = 50 m)., (**a**) Composite model x-component, (**b**) Hybrid model x-component, (**c**) Composite model y-component, (**d**) Hybrid model y-component, (**e**) Composite model z-component (**f**) Hybrid model z-component, (**g**) Composite model magnetic total field modulus and (**h**) Hybrid model magnetic total field modulus.

**Figure 9:**
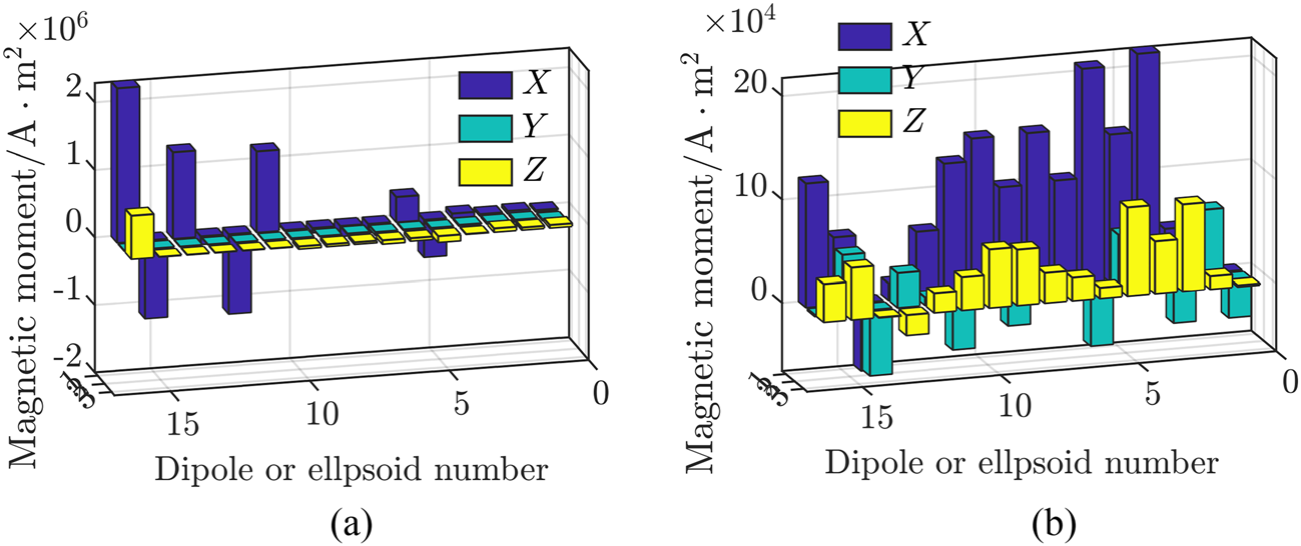
Magnetic moments solved based on the TSVD-GCV algorithm. (**a**) Composite model and (**b**) Hybrid model.

**Figure 10:**
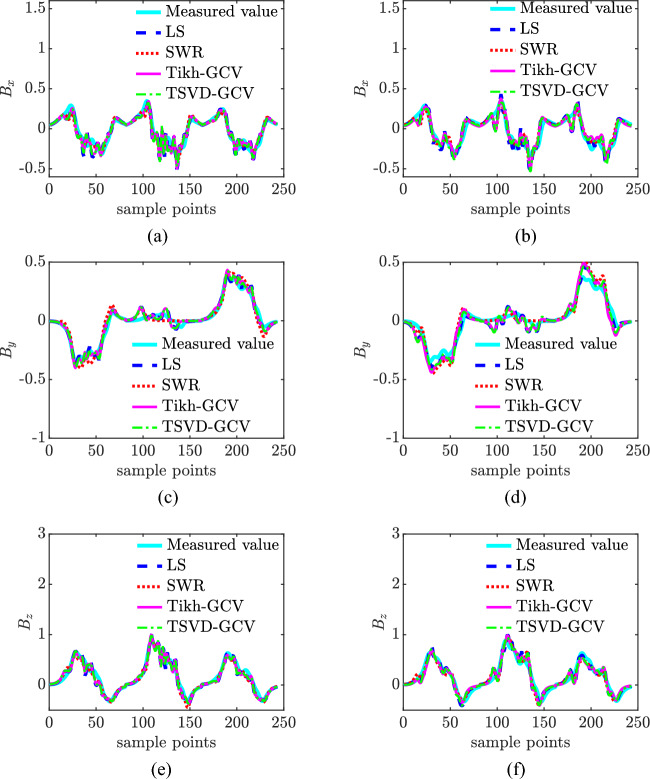
Magnetic field fitting curve(SNR = 0 dB, *z* = 17.2 m). (**a**) Composite model x-component, (**b**) Hybrid model x-component, (**c**) Composite model y-component, (**d**) Hybrid model y-component, (**e**) Composite model z-component and (**f**) Hybrid model z-component

**Figure 11:**
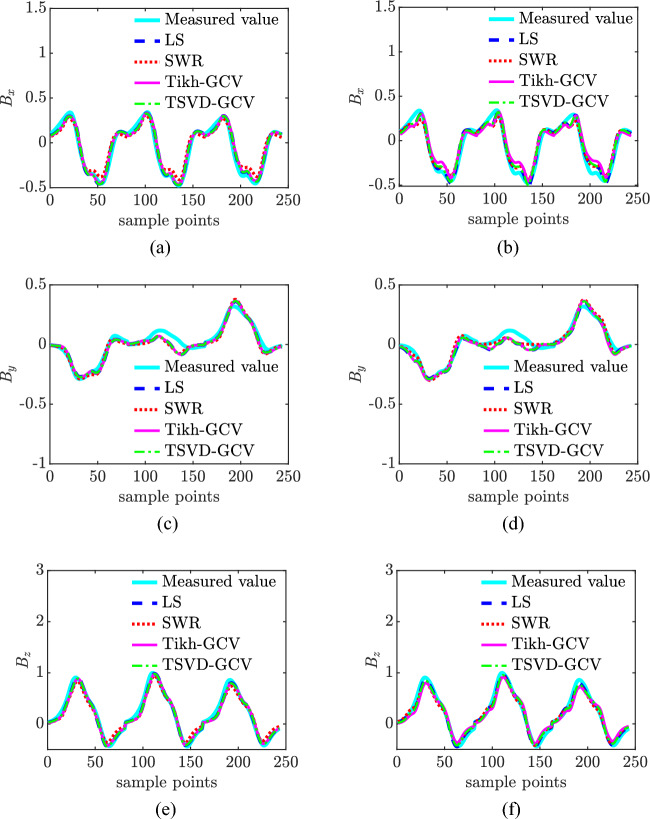
Magnetic field extrapolation curves(SNR = 0 dB, *z* = 17.2 m → 28.8 m). (**a**) Composite model x-component, (**b**) Hybrid model x-component, (**c**) Composite model y-component, (**d**) Hybrid model y-component, (**e**) Composite model z-component and (**f**) Hybrid model z-component.

The LS algorithm, stepwise regression method, Tikhonov-GCV, and TSVD-GCV algorithms were used to solve the magnetic moments, respectively. In performing the magnetic moment solution for the ship model, the observed magnetic field data were measured at a depth of 1 time the ship width and extrapolated to a depth of 2 times the ship width. The magnetic field measurement data under different signal-to-noise ratio conditions are used for model validation, and the noise is Gaussian white noise. The number of coefficient matrix conditions is shown in Table 3, from which it can be seen that the number of coefficient matrix conditions of the improved composite model is only 0.2091 of the number of coefficient matrix conditions of the hybrid model, from which it can be seen that the degree of pathology of the equation set of the improved composite model is much smaller than that of the equation set of the hybrid model, and thus the robustness of the solution of the magnetic moments is stronger. The relative error of magnetic field and the relative error of magnetic field extrapolation are shown in Table 4 and Fig. 6. Combining the graphs, it can be seen that the modeling accuracies of the two models under different SNR conditions with different magnetic moment solving algorithms are not much different, with the maximum difference of about 3%, and the relative error of magnetic field fitting and the relative error of magnetic field extrapolation of the composite model under high SNR conditions are both within 10%, and the relative error of magnetic field fitting can also be reached to 3% under 0 dB SNR conditions, and the relative error of magnetic field extrapolation can also be reached to 4% under 0 dB SNR conditions. The relative errors of magnetic field fitting and magnetic field extrapolation are within 30% and 15% even at 0 dB SNR. The CPU computation time is shown in Table 5 and Fig. 7. Combined with the graphs, it can be seen that the computation time of the composite model is less than that of the hybrid model when solving magnetic moments with the four algorithms, which indicates that the computational complexity of the composite model is less than that of the hybrid model. At 0 dB SNR, the depth of solving is 50 m. The depth of the composite model is 50 m, and the depth of the composite model is 50 m. The depth of the composite model is 50 m. When the SNR is 0 dB, the spatial distribution of the magnetic field at a depth of 50 m based on the TSVD-GCV algorithm is shown in Fig. 8, from which it can be seen that the spatial distribution of the magnetic field is basically the same for the two models, which suggests that the composite model is able to realize the effective modeling of the ship's magnetic field. The magnetic moments solved based on the TSVD-GCV algorithm are shown in Fig. 9, from which it can be seen that the magnetic moments of the two models are quite different. The magnetic field fitting curve (SNR = 0 dB, z = 17.2 m) is shown in Fig. 10, and the magnetic field extrapolation curve (SNR = 0 dB, z = 17.2 m → 28.8 m) is shown in Fig. 11. From the magnetic field fitting curves and extrapolation curves, the composite model still has a high fitting accuracy for the ship's magnetic field under low SNR conditions.

**Table 3:** Coefficient matrix condition number.

Model	Coefficient matrix condition number
Hybrid	7.1004e3
Composite	**1.4846e3**

Significant values are in [bold].

**Table 4:** Relative error of magnetic field fitting and relative error of magnetic field extrapolation.

Model	Algorithm	Error	SNR/dB					
			0	10	20	30	40	50
Hybrid model	LS	*E*_1_	0.3010	0.1154	0.0750	0.0695	0.0690	0.0689
		*E*_2_	0.1456	0.0932	0.0856	0.0843	0.0842	0.0842
	SWR	*E*_1_	0.2518	0.1123	0.0762	0.0707	0.0701	0.0700
		*E*_2_	0.1710	0.0953	0.0859	0.0847	0.0848	0.0849
	Tikhonov-GCV	*E*_1_	0.2698	0.1127	0.0750	0.0696	0.0690	0.0690
		*E*_2_	0.1732	0.0974	0.0860	0.0844	0.0842	0.0843
	TSVD-GCV	*E*_1_	0.2797	0.1124	0.0753	0.0697	0.0690	0.0689
		*E*_2_	0.1476	0.1476	0.0857	0.0844	0.0842	0.0842
Composite model	LS	*E*_1_	0.2818	0.1304	0.1030	0.0997	0.0994	0.0993
		*E*_2_	0.1476	0.0960	0.0898	0.0896	0.0893	0.0893
	SWR	*E*_1_	0.2554	0.1300	0.1044	0.1013	0.1009	0.1008
		*E*_2_	0.1808	0.0998	0.0909	0.0904	0.0899	0.0899
	Tikhonov-GCV	*E*_1_	0.2741	0.1294	0.1030	0.0997	0.0994	0.0994
		*E*_2_	0.1572	0.0976	0.0904	0.0899	0.0896	0.0896
	TSVD-GCV	*E*_1_	0.2791	0.1291	0.1029	0.0998	0.0995	0.0995
		*E*_2_	0.1509	0.0961	0.0899	0.0896	0.0894	0.0894

**Table 5:** CPU computation time.

Model	Algorithm	Computation time/s
Hybrid model	LS	8.0112e-04
	SWR	0.0029
	Tikhonov-GCV	0.0225
	TSVD-GCV	0.0208
Composite model	LS	**5.8042e−04**
	SWR	**0.0019**
	Tikhonov-GCV	**0.0223**
	TSVD-GCV	**0.0206**

Significant values are in [bold].

In summary, the comparison of the two model indicators is shown in Table 6. From the four evaluation indexes, the composite model is comparable to the hybrid model in terms of the relative error of magnetic field fitting and the relative error of magnetic field extrapolation, and is able to achieve higher modeling accuracy. In terms of the number of coefficient matrix conditions, the composite model is smaller than the hybrid model, and the magnetic moment solving is more robust. In terms of computational complexity, the composite model has a smaller computational degree, which is more favorable to engineering practice. Therefore, the composite model can be used for modeling the magnetic field of ships, and compared with the hybrid model, the computational speed is improved on the basis of retaining higher modeling accuracy.

**Table 6:** Comparison of model indicators.

Model	Coefficient matrix condition number	Relative error of magnetic field fitting	Relative error of magnetic field extrapolation	Computational complexity
Hybrid	High	High	High	High
Composite	**Low**	**High**	**High**	**Low**

Significant values are in [bold].

## Conclusion

The improved ellipsoid and magnetic dipole array composite model proposed in this paper has the following advantages over the traditional hybrid model:

1. While maintaining a high magnetic field modeling accuracy, the number of coefficient matrix conditions is effectively reduced, which improves the model robustness.
2. The model complexity is reduced, effectively reducing the computational complexity, which is conducive to engineering applications.
3. The composite model can be solved by a variety of regularization methods, and all of them can achieve high modeling accuracy, and the relative error of the magnetic field fitting and the magnetic field extrapolation error under high signal-to-noise ratio conditions are less than 0.1.
4. The ship magnetic field modeling method adopted in this paper can effectively counter Gaussian noise interference, and the relative error of magnetic field fitting is less than 0.3 and the relative error of magnetic field extrapolation is less than 0.15 under the condition of 0 dB signal-to-noise ratio.

## Data Availability

The datasets used and analyzed during the current study available from the corresponding author on reasonable request.
